# A meta-analysis on anticoagulation after vascular trauma

**DOI:** 10.1007/s00068-020-01321-4

**Published:** 2020-02-17

**Authors:** Shujhat Khan, Hussein Elghazaly, Areeb Mian, Mansoor Khan

**Affiliations:** 1 Department of Surgery, Imperial College London, London, UK; 2 St Mary’s Hospital, Praed Street, London, UK

**Keywords:** Vascular surgery, Trauma, Anticoagulants

## Abstract

**Purpose:**

There is much debate regarding the use of anticoagulation following vascular trauma. The aim of this meta-analysis was to compare the outcome of trauma following administration of anticoagulation medication.

**Methods:**

The literature search was carried out using Ovid MEDLINE and PubMed databases to search for keywords and MeSH terms including “Anticoagulation”, “Vascular Surgery”, “Vascular Trauma”, “Vascular Repair”, “Repair” and “Wounds and Injuries”.

**Results:**

Use of anticoagulation was associated with a better prognosis for overall vascular trauma outcomes (weighted OR 0.46; 95% CI 0.34–0.64; *P* < 0.00001), as well as reduced risk of amputation for both lower and upper limb vascular trauma (weighted OR 0.42; 95% CI 0.22–0.78; *P* = 0.007), and reduced occurrence of reoperation events and amputations in isolated lower limb vascular trauma (weighted OR 0.27; 95% CI 0.14–0.52; *P* < 0.0001).

**Conclusion:**

There was a statistically significant correlation between the use of anticoagulation and vascular trauma outcome. A major limitation with many of the studies includes a lack of prospective analysis and therefore we recommend prospective studies to properly elucidate prognostic outcomes following use of these anticoagulants. Further studies need to be conducted to assess the effects of timing of anticoagulant delivery, dosages and severity of traumatic injury. Thus, this would prove to be very useful in the formation of guidelines.

## Introduction

The usage of anticoagulation in vascular surgery following trauma remains controversial due to the potential increased risk of local and systemic haemorrhage [1]. Conversely, during elective arterial repair, intraoperative systemic anticoagulation (ISA) is routinely provided in operations such as arterial reconstruction and peripheral artery disease (PAD) [2–5].

There is however a lack of randomised controlled trials demonstrating its optimal use in the context of vascular trauma. As a result, surgeons are hesitant to provide intravenous ISA to patients in this setting due to concern for potential local and systemic bleeding. Additionally, anticoagulants have been shown to improve arterial patency in elective vascular surgery. However, the translatability of this evidence to traumatic vascular injury repair remains questionable. By analysing different studies, we hope to see whether the use of ISA has similar benefits to those seen in elective peripheral vascular surgery.

The mechanisms for anticoagulants are well described in the literature and are widely used for a spectrum of conditions [6–14]. Ever since its discovery, heparin has been an essential element in elective peripheral vascular surgery. Previously, thrombus formation occurred along suture lines due to the disruption of laminar flow, stasis and release of procoagulant factors such as tissue thromboplastin. This proved to be a major contributing factor in arterial reconstruction failure. The discovery of heparin in the early twentieth century allowed these effects to be countered and thus has been shown to significantly improve arterial patency and overall reconstruction success for procedures such as arterial bypass grafting in peripheral vascular disease. However, for vascular trauma surgeons, managing these patients remains a delicate balance and no consensus has yet been achieved regarding the use of anticoagulation medication in surgical repair of traumatic extremity arterial and venous injuries. This is evidenced by the lack of guidelines surrounding the use of ISA in these cases.

This meta-analysis explores current practice in the use of intraoperative anticoagulant treatments in vascular trauma surgery and assesses whether the use of these is beneficial to this patient group. This includes analysing whether there is improved arterial patency, limb salvage and reduced occurrence of venous thromboembolisms following anticoagulation use.

## Methods

### Search strategy

**Fig. 1:**
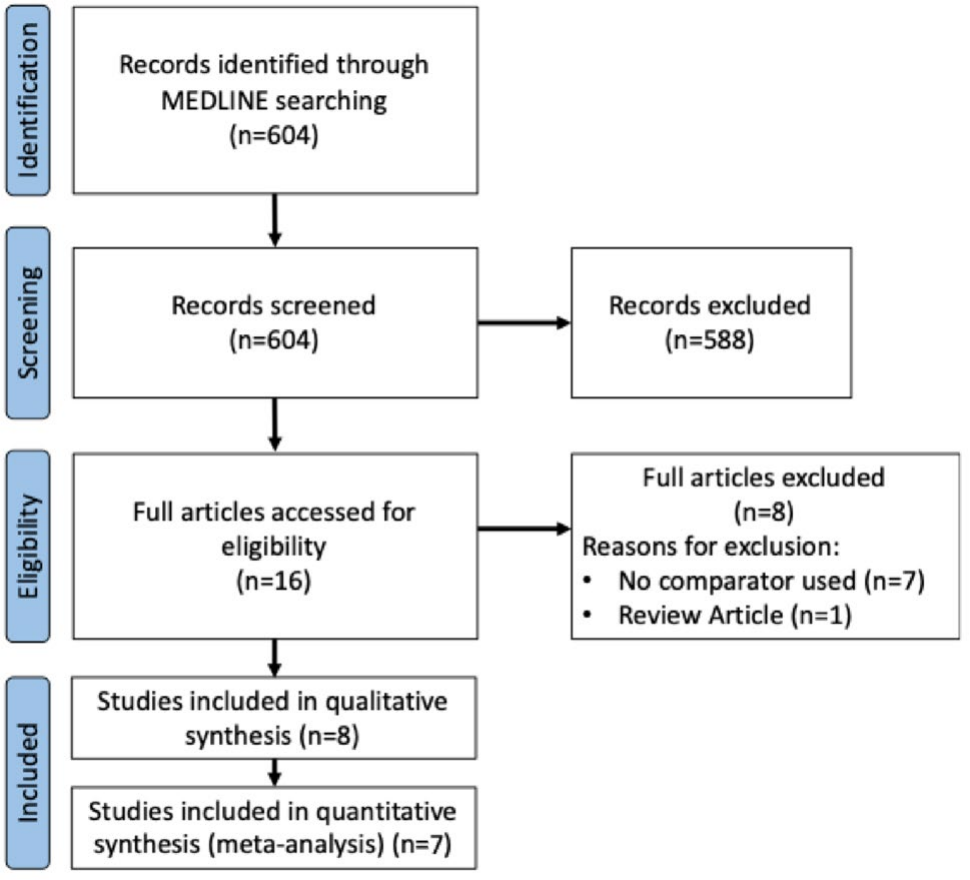
Flow chart explaining stepwise selection procedure and reasons for exclusion of studies

A literature review was conducted based on the Preferred Reporting Items for Systematic Reviews and Meta-Analysis guidelines (PRISMA) [15, 16]. A search was conducted for studies comparing anticoagulation therapies compared to no treatment in the context of vascular trauma. The MEDLINE database was searched using Ovid, in addition to PubMed for keywords and MeSH terms including “Anticoagulation”, “Vascular Surgery”, “Vascular Trauma”, “Vascular Repair”, “Repair” and “Wounds and Injuries”. The full search strategy can be seen in Fig. 1. Additionally, we checked the reference lists of relevant publications for any unidentified trials.

## Study types

All studies were published in English. They all focussed on vascular trauma in adults. Six studies included amputation, repair thrombosis, and reoperation rate data. Two studies included data regarding arterial patency failure and venous thrombo-embolism (VTE) development. All studies included were retrospective in nature and there were no randomised control trials.

### Inclusion and exclusion criteria

All clinical studies investigating the effects of anticoagulation in the context of vascular trauma were included. All trials irrespective of their prospective or retrospective nature were included. No language or date restrictions were applied. Only human studies were considered and all studies reporting results from animal or in vitro models were excluded.

All studies of the use of perioperative anticoagulation were included in this meta-analysis; this includes pre-operative and intra-operative anticoagulation therapies.

### Risk of bias assessment

The risk of bias using the Cochrane Handbook for Systematic Reviews of Interventions [17]. The following risk of bias domains was assessed: random sequence generation; allocation concealment; blinding of participants and personnel; blinding of outcome assessment; incomplete outcome data; selective outcome reporting; funding bias; and other risks of bias.

### Statistical analysis

Various effect size estimators such as correlation coefficient, risk ratio, and standardised mean difference were analysed. It was determined an odds ratio (OR) would the most efficient effect size estimator due to the dichotomous nature of the data. 95% confidence intervals were also calculated for each effect size. Inverse variance within each study and between studies was used to the weight the corresponding effect sizes. It was primarily determined by the sample size. The significance of variability among effect sizes (heterogeneity) was assessed by computing the *Q* statistic and *I*-square index. *I*-squared statistic was also computed which describes the percentage variation across studies that is due to heterogeneity rather than chance, which unlike *Q*, is not inherently dependent upon the number of studies considered [18].

## Results

### Study characteristics

A total of 604 citations were identified from the literature search. Eight clinical studies involving a total of 1807 patients met the inclusion criteria for this review whilst seven clinical studies involving a total of 1383 patients were included for the statistical analysis. All studies included were retrospective in nature and there were no randomised control trials. The remaining studies were excluded for various reasons, as depicted in Fig. 1.

All studies were published in English between 1988 and 2017 whilst the sample size varied from 99 to 435 patients per study. Details of the methodological assessment are depicted in Table 1.

**Table 1:** Characteristics of studies

Study	Time of delivery	Outcomes assessed	Location
Loja et al. [28]	ISA	Repair thrombosis and/or limb amputation	Lower
Frank et al. [26]	ISA and post operative	VTE	No distinction
Maher et al. [27]	ISA	Arterial patency	Upper and lower
Humphries et al. [29]	ISA	Amputation and repair failure	No distinction
Wang et al. [24]	ISA/ILA and post operative	Amputation, arterial patency, VTE	No distinction
Guerrero et al. [23]	ISA/recovery room	Amputation	Lower
Melton et al. [25]	ISA	Amputation	Lower
Wagner et al. [22]	ISA	Amputation	Lower

*ISA* intraoperative systemic anticoagulation, *ILA* intraoperative local anticoagulation

The majority of patients were males, representative of the increased incidence of vascular trauma in this population [19]. The studies included a range of trauma aetiologies: accident from vehicles, falls, knee hyperextension, gunshots, penetrating stab wounds, and explosions. Moreover, the studies looked at a range of different locations that were affected by the trauma. There were 4 studies that exclusively looked at lower limb trauma [20–23] whilst 3 looked at both upper and lower [24–26]. However, one study did not make clear distinctions between anatomical locations of the traumatic insult [27]. It would appear that the majority of studies looked at included patients suffering from vascular trauma alongside associated fractures although in some studies, it was difficult to determine the exact injuries. However, in two of the studies, they included patients suffering from head injuries with vascular trauma and fractures [21, 24] but it is unclear if there was a difference in treatment protocol and outcomes with these patients alone. Notably, there were no identifiable studies on the effect of solely administering anti-platelet therapy on the outcomes of vascular trauma surgery. Seven studies reported the use of anticoagulation alone in patients with vascular trauma [20, 21, 23–27] and one study reported the effects of anticoagulation and anti-platelet (aspirin) therapy in this context [22]. No studies looking at the use of anti-platelets alone were found. Moreover, there was only one study assessing outcomes following post-operative anticoagulation [22], and one study evaluated the use of perioperative anticoagulation [20], whilst 6 studies assessed the prognosis of intraoperative anticoagulation [21, 23–27].

#### Studies demonstrating positive outcomes

Four studies were identified [20, 21, 24, 25] that demonstrated positive outcomes with the use of anticoagulants in the context of vascular trauma. Negative outcomes were classified as failure to maintain arterial patency, development of venous thromboembolism and amputation, whilst positive outcomes were considered to be a reduction in the incidence of adverse intraoperative and post-operative complications. All four studies retrospectively analysed patient data, whilst three of the studies included patients from two or more level-one trauma centres in their retrospective analysis [21, 24, 25].

To the author’s knowledge, Wagner et al. [20] published the first study pertaining to the use of anticoagulants in patients with vascular trauma. It is worth noting, however, that this study only included one patient with no anticoagulation therapy; the remaining patients received local, rather than systemic, anticoagulation. In conducting this meta-analysis, we made the assumption that no heparin was administered in the case of locally infused heparin. The results showed systemic anticoagulation is significantly better than local anticoagulation infusion and leads to increased limb salvage with greater arterial patency rates. Similarly, Guerrero et al. and Maher et al. showed anticoagulation improved limb salvage in patients who present with arterial injuries in the common iliac and popliteal arteries who then underwent operative intervention [21, 25]. Furthermore, patients who were administered ISA had a decreased return to the operating theatre for post-operative bleeding and were significantly more likely to maintain arterial patency, in addition to requiring a shorter Intensive Care Unit and overall stay in the hospital [25].

Conversely, ISA in patients with major vascular injury of the neck, torso or proximal upper (to the elbow), and lower (to the knee) extremities was shown to reduce the incidence of VTE and subsequent post-operative addition of enoxaparin prophylaxis also reduced VTE incidence [24]. The authors also revealed that venous injury was an independent predictor of VTE and as such, patients with major venous injuries are at high risk for VTE, regardless of intraoperative management.

#### Studies demonstrating neutral outcomes

Four studies [22, 23, 26, 27] reported no statistically significant difference in outcomes with the use of anticoagulants in patients with vascular trauma, and thus do not routinely recommend anticoagulation therapy. These studies also retrospectively analysed outcomes. Additionally, those who received systemic intraoperative anticoagulation had a lower Injury Severity Score compared with patients who did not, with a trend indicating a worse postoperative outcome following anticoagulation use although this was not statistically significant [27]. However, there is a discrepancy between intra-operative complications with data suggesting no heparin-related bleeding complications occurring [23, 27] whilst other studies suggest ISA was associated with an increase in blood product requirements and prolonged hospital stay [26].

Additionally, there does not appear to be a statistically significant difference in the rate of haemorrhage, thrombosis, cerebral vascular accident, limb amputation, compartment syndrome and mortality following postoperative heparin use compared with postoperative aspirin for five consecutive days [22].

## Results of the meta-analysis

### Overall outcome of vascular trauma with anticoagulation

**Fig. 2:**
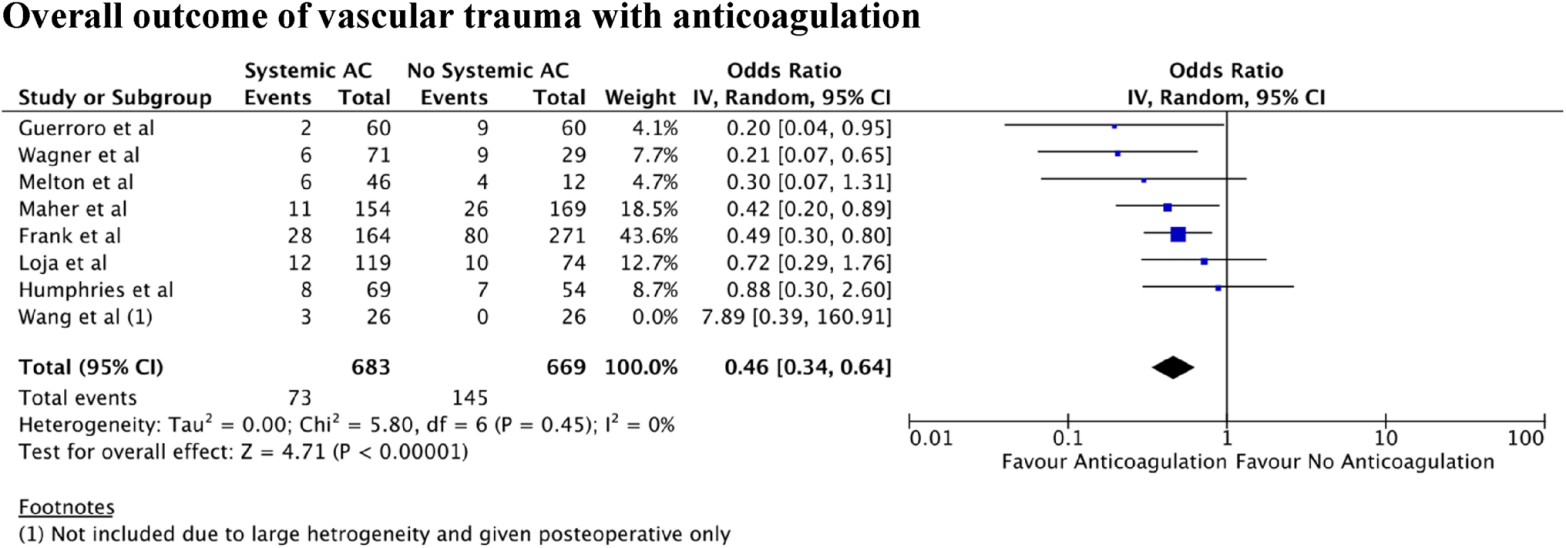
Positive and negative outcomes in the vascular trauma looking at anticoagulant use

A total of 7 independent study samples, which included a total of 1352 participants, met the inclusion criteria (Fig. 1). The studies utilised a variety of measures, methods, intended outcomes, and analytical approaches. There was low evidence of statistical heterogeneity between these studies (*I*^2^ = 0%). Meta-analysis (Fig. 2) revealed a significant association between systemic anticoagulation use and reduction in composite negative outcomes including amputation, repair thrombosis, reoperation, and VTE occurrence (weighted OR 0.46; 95% CI 0.34–0.64; *P* < 0.00001).

### Amputations

**Fig. 3:**
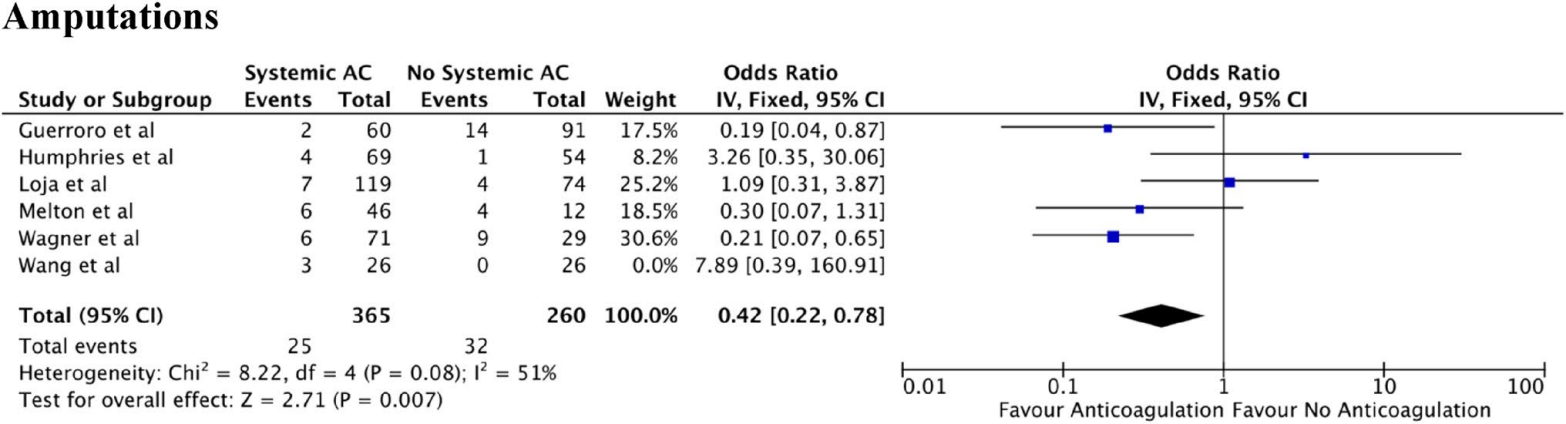
Amputations in vascular trauma looking at anticoagulant use

Six studies compared the effect of anticoagulant use in vascular trauma and looked specifically at the number of amputations (Fig. 3). The total number of participants in these studies was 625. However, the study presented by Wang et al. [23] was excluded in the meta-analysis due to the extremely small n value. There was evidence of statistical heterogeneity between these studies (*I*^2^ = 51%). Meta-Analysis showed that patients in the anticoagulation arm had a lower amputation rate compared to those who were not given intraoperative anticoagulation (weighted OR 0.42; 95% CI 0.22–0.78; *P* = 0.007).

### Lower limb vascular trauma

**Fig. 4:**
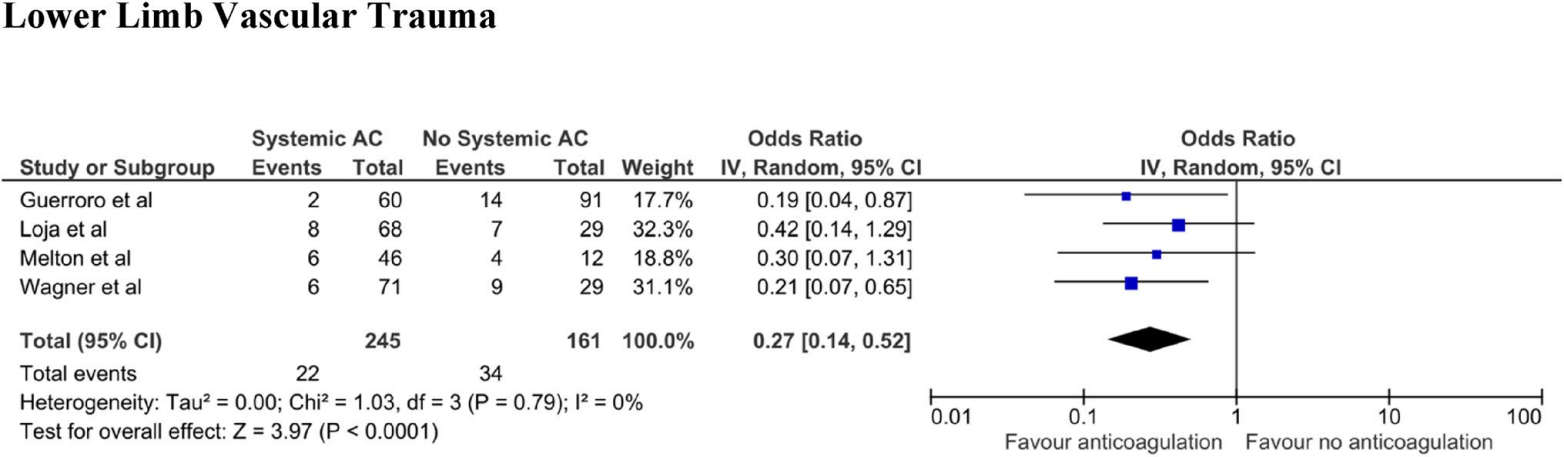
Negative/positive outcomes in lower limb vascular trauma looking at anticoagulant use

Four studies had compared the effect of systemic anticoagulant use in lower limb vascular trauma (Fig. 4). The total number of patients across these studies was 406. The studies looked at negative outcomes including amputation and reoperation events. There was no evidence of statistical heterogeneity between these studies (*I*^2^ = 0%). The random-effects model showed patients in the anticoagulation arm had lower composite negative outcomes compared to those who were not given intraoperative coagulation (weighted OR 0.27; 95% CI 0.14–0.52; *P* < 0.0001).

## Discussion

No meta-analysis assessing the use of anticoagulants or anti-platelets in the context of vascular trauma has previously been conducted. Indeed, medication that alters the natural coagulation pathway can often pose a challenge to vascular surgeons, particular in traumatic scenarios. The studies analysed in this meta-analysis drew contrasting conclusions with several papers suggesting such pharmacological use is beneficial in minimising distal and small vessel thrombosis and thereby improving arterial patency [20, 21, 24, 25, 28] whilst other studies indicating no difference in patient outcomes [22, 23, 26, 27]**.** However, the overall analysis of the statistical results indicates that the use of anticoagulation in vascular trauma is associated with an improved prognosis for the patient and a reduced rate of amputations (Fig. 3). Perhaps of note, none of the groups suggested an increase in negative outcomes from the pharmacological use of anticoagulation. In addition, systemic anticoagulation, of which heparin was the most widely used anticoagulant, is shown to have a greater beneficial effect on outcomes compared to local administration, especially in major operations [20].

All the studies used heparin as their choice of anticoagulation whilst only one study assessed the impact of enoxaparin [24]. Whilst the majority of studies reported use of anticoagulation following vascular trauma, only one study in literature was found to have examined anti-platelets. No studies combined anticoagulation with anti-platelet therapy. This was perhaps due to the increased risk of bleeding such therapy poses. The use of anti-platelets alone has been associated with increasing the risk of post-traumatic bleeding and morbidity [29, 30] and therefore, the risk is largely dependent on platelet function that is perhaps best assessed by determining overall clot strength through a whole blood assay [31]. In a time-restricted situation such as in traumatic vascular surgery, this can be a challenging feat. Interestingly, this contrast with guidelines suggesting the potential benefit that anti-platelets can have in an elective surgery cases, is perhaps due to the altered haematological physiology in those suffering from trauma [32–35]. As such, the use of anti-platelets is not significantly beneficial in the context of traumatic vascular surgery. Wang et al. [22] analysed anti-platelet use and found there to be no difference in outcomes. However, their sample size was too small to draw any significant conclusion. This limitation, along with a lack of studies found in the literature means that statistically, the impact on patient outcomes with anti-platelet use in vascular trauma remains unclear.

In addition, the varied protocols between the included studies merits further discussion. Notably, the majority of the included studies were retrospective in nature, which is understandable given the critical condition of the included population. No clear study defined protocols were evident regarding the rationale for or against anticoagulation, which was mostly down to the clinician’s discretion. Therefore, unmeasured study biases may exist. Furthermore, this is also the case with regards to the choice of intraoperative versus post-operative anticoagulation. As such, we cannot ascertain whether there was an intrinsic difference in the clinical presentation of the different study groups; this remains a significant limitation of the existing literature which doesn’t provide a rationale for the determination of whether intra-operative or post-operative anticoagulation was used.

Anticoagulants, on the other hand, were shown to be beneficial in reducing complication rates following traumatic vascular injuries. Furthermore, heparin was the most widely used anticoagulant in these studies. Because of the versatility of heparin use in vascular surgery, it is a mainstay in vascular surgery. It has the benefit of providing immediate results, the ability to irrigate directly into tissue graft and vessels as well as the fundamental ability to reverse its action quickly using protamine. However, to reduce the risk of heparin-induced thrombocytopenia following vascular traumatic surgery, unfractionated heparin such as certoparin can instead be used to provide a more beneficial prognosis for major trauma [36, 37].

However, it is important to note that whilst two of the studies included patients with cerebral trauma, the specific details about these were not explained. Indeed, it would appear that in the majority of mentioned studies, the decision to use ISA was at the surgeon’s discretion, and not on the basis of a study-defined protocol. As such, recommending the use of anticoagulation for cerebral vascular injury or other non-vascular injuries concurrently is outside the scope of this review. In addition, the studies reveal anticoagulation is more beneficial in lower limb trauma (Fig. 4). This is especially significant considering the worse prognosis that lower limbs tend to have compared with upper limb complex trauma [38]. The reasons for this discrepancy between upper limb and lower limb outcomes remains unclear; and there is currently insufficient data to ascertain whether this discrepancy is due to an inherent difference in the response of upper and lower limb traumatic events to anticoagulation, or whether confounding variables may influence this finding. Notably, lower limb injuries are often associated with more extensive bleeding and concomitant injuries; thus, confounders include the extent of injury and bleeding as well as the presence of concomitant injuries [39]. Therefore, we cannot ascertain whether the site of injury is a predictor of the response to anticoagulation, or whether confounding variables may have influenced these outcomes.

The effects of traumatic injury on inducing coagulopathy have been well documented [40–42] and result in a higher rate of tissue death and organ failure, ultimately contributing to worsened morbidity and mortality [43, 44]. As such, the use of anticoagulants in trauma-induced coagulopathy may be harmful due to worsening of the coagulopathic state. Therefore, sound clinical assessment of the patient is necessary to appropriately decide whether or not anticoagulants can be used. The studies we analysed suggested a lack of iatrogenic harm with ISA use. However, these patients were unlikely to be severely coagulopathic in the first place. Interestingly, a number of studies reported no significant difference in the use of anticoagulation against no anticoagulation [22, 23, 26, 27]. This is reflective of the complicated nature of coagulation following trauma with multiple factors influencing the severity of coagulopathy. This includes hypothermia, hypoperfusion, acidaemia, and consumption of coagulation factors, which, along with the patient’s genetic background, the severity of traumatic injury, co-morbidities, medication and pre-hospital treatment can lead to a complicated scenario where optimal treatment regime can become complex [45, 46].

One of the main arguments against the use of anticoagulation in the context of vascular trauma has been that it may increase the risk of haemorrhage related complications from commitment injuries. In several studies the use of anticoagulation in arterial injury repair without contraindications has not been shown to significantly increase haemorrhagic complications [2, 21, 27] However, Loja et al. did report that in their population there was an increase in blood product usage in patients who received ISA [26]. It should be noted that the majority of patients who did not receive anticoagulation generally had a lower injury severity score (ISS) compared to those who did. A lower ISS is consistent with more isolated injuries. Therefore, this meant that patients who received ISA would likely have had a lower risk of bleeding complications in the first place. This highlights the need for larger studies with ISS matching to definitively determine the relationship between ISA use and bleeding complications.

This meta-analysis has demonstrated that use of systemic anticoagulants in the context of vascular trauma is significantly associated with a reduction in the rate of arterial patency failure, and amputation rates, as well as reduced risk of VTE development. We thereby cautiously recommend the use of anticoagulants in the general context of vascular trauma for patients with no existing contraindications.

## Limitations

It is, however, important to consider the limitations of the studies. Notably, none of the studies in this meta-analysis originate from high-grade RCT and there is a strong risk of bias, especially regarding randomisation and allocation concealment. Numerous additional study limitations are also noteworthy. Take for example the results from Wagner et al. [20]. These were confined to popliteal artery injuries and therefore caution is strongly suggested before generalising the data to all vascular injuries. Furthermore, Frank et al.’s study [24] lacks the inclusion of perioperative in the multivariate analysis, and thromboelastography was not used to aid clinical decision making, as employed at many trauma centres. Maher et al. [25] are limited by the fact that the decision to use ISA was at the surgeon’s discretion, and not on the basis of a study-defined protocol. Guerrero et al.’s [21] major limitation is the lack of differentiation between intraoperative and postoperative anticoagulation, an important consideration as different protocols may influence the results. The majority of studies had mixed injuries, including patients with injuries to brachial, axillary, femoral, popliteal and iliac arteries, in which failure of arterial patency is not the only variable affecting amputations. Finally, factors such as degree of soft tissue injury, infection, co-morbidities, the occurrence of concomitant injuries, and tranexamic acid usage were not captured in all these retrospective studies and therefore may further confound findings. Combined, these factors limit the strength of the evidence for our conclusion. The lack of quality studies, the difference in mode of anticoagulant delivery, location of injury and outcomes of interest including the overall pattern of injury or ISS, the condition of the patient on presentation, and finally differences in the extent or details of the repair, all suggest that we are unable to conclusively form a strong opinion. As such, whilst the studies appear to suggest a superiority of anticoagulation, we recommend further studies be done to allow better comparison to elucidate a better understanding about the use of anticoagulants on traumatic vascular surgery.

## Conclusion

The results of this meta-analysis suggest anticoagulation may have some benefit in vascular trauma surgery of the peripheral extremity to reduce intraoperative and postoperative complications. No studies demonstrated an increase in negative outcomes with anticoagulation use. Despite the improvement in limb salvage and reduction in complications seen with anticoagulation, its safe use, still relies on wise clinical judgement and use in the appropriate patient group. Indeed, in such patients use of thromboelastrography (ROTEM) which is already widely used in clinical practice, should continue to be maintained.

It is clear that there is a great need for higher quality controlled prospective studies to be conducted to assess the various factors that can affect prognosis. This includes the timing of anticoagulants, dosages and the severity of traumatic injury. This would further the evidentiary base allowing the development of guidelines concerning ISA use, thereby permitting vascular surgeons to make decisions that will better aid in achieving a more favourable prognosis.
